# Expanding Bicycle-Sharing Systems: Lessons Learnt from an Analysis of Usage

**DOI:** 10.1371/journal.pone.0168604

**Published:** 2016-12-15

**Authors:** Ying Zhang, Tom Thomas, M. J. G. Brussel, M. F. A. M. van Maarseveen

**Affiliations:** 1 Faculty of Geo-Information Science and Earth Observation (ITC), University of Twente, Enschede, The Netherlands; 2 Centre for Transport studies, University of Twente, Enschede, The Netherlands; Peking University, CHINA

## Abstract

Bike-sharing programs, with initiatives to increase bike use and improve accessibility of urban transit, have received increasing attention in growing number of cities across the world. The latest generation of bike-sharing systems has employed smart card technology that produces station-based data or trip-level data. This facilitates the studies of the practical use of these systems. However, few studies have paid attention to the changes in users and system usage over the years, as well as the impact of system expansion on its usage. Monitoring the changes of system usage over years enables the identification of system performance and can serve as an input for improving the location-allocation of stations. The objective of this study is to explore the impact of the expansion of a bicycle-sharing system on the usage of the system. This was conducted for a bicycle-sharing system in Zhongshan (China), using operational usage data of different years following system expansion. To this end, we performed statistical and spatial analyses to examine the changes in both users and system usage between before and after the system expansion. The findings show that there is a big variation in users and aggregate usage following the system expansion. However, the trend in spatial distribution of demand shows no substantial difference over the years, i.e. the same high-demand and low-demand areas appear. There are decreases in demand for some old stations over the years, which can be attributed to either the negative performance of the system or the competition of nearby new stations. Expanding the system not only extends the original users’ ability to reach new areas but also attracts new users to use bike-sharing systems. In the conclusions, we present and discuss the findings, and offer recommendations for the further expansion of system.

## 1. Introduction

Cycling is widely associated with benefits in terms of the environment, society, and economy [1,2]. The combined use of a bicycle and public transport for a trip, which has been regarded as part of the solution for achieving a more sustainable transport, has grown over the past few years [3,4]. Recently, bicycle-sharing programs, with initiatives to increase bike use and improve “the last mile” of urban transit, have received increasing attention in more and more cities across the world [5,6]. Published studies have shown that for both utilitarian and recreational purposes, cycling has increased in some cities that are operating bicycle-sharing systems [7,8]. Currently, more than 600 such systems are operating around the world, and many systems are being planned and will start operation in the near future [9].

The latest generation of bicycle-sharing systems has employed smart card technology, which enables users to monitor the number of available bikes and parking slots via real-time online maps or mobile apps [6,8]. This technology produces station-based data or trip-level data, which facilitates studies of the practical use of bicycle-sharing systems [10]. Some studies have employed data mining techniques [11–14] and visualization techniques [15–17] to uncover the spatial and temporal patterns of cycle trips. Other studies have explored bike-sharing use, in terms of its impact on other transport [18–20], user demographics [21,22], and the influence of built environment factors [23–32], weather and calendar events [33,34] on shared bike demand. Most of the aforementioned studies, except one from Goodman and Cheshire [21], did not address the changes in usage (i.e. in terms of both users and demand) over the years, and did not study the impact of the system expansion on its performance. However, system usage might not be stationary, and may change over the years. Examining changes in usage of a system over the years enables the identification of factors that influence the system’s performance, and can also serve as an input for improving the location-allocation of stations and planning for new stations.

In this context, the objective of this study is to explore the changes of system usage over the years and impact of the expansion of a bicycle-sharing system on the usage of the system. This study was conducted for a bicycle-sharing system in Zhongshan (China), using trip data from March 2012, March 2013, and March 2014. Such a system gradually expanded the number of stations equipped with parking slots between March 2012 and March 2013, and again between March 2013 and March 2014. This study contributes to the understanding of how the system usage (in terms of both users and demand) changes following the expansion of the system. Moreover, this study also provides insights into: (1) what information can be extracted from trip data to evaluate the impact of system expansion on its use; and (2) what can be learnt from this evaluation to promote the further extension of the system.

The remainder of this paper is organized as follows. The next section provides a literature review. Section 3 introduces the study area and data preparation, and explains the research method. Section 4 presents and discusses the results. Finally, Section 5 draws the key conclusions and offers the recommendations for the system expansion and further study.

## 2. Literature review

With the availability of open data, i.e. station-based data or trip level data, a large number of studies have been carried out to explore the practical usage of bicycle-sharing systems. In general, those studies mainly cover four aspects: Firstly, to explore the spatial and temporal patterns of bike use over the time of day, using data mining [12–14] and visualization [15–17] techniques. Froehlich et al. [12] grouped stations based on bicycle activity at the stations of Barcelona’s public bike system, and Kaltenbrunner et al. [13] extended the former analysis by predicting bicycle activity at Barcelona’s stations over the hours of the day. Vogel et al. [14] examined activity patterns of bike use at the stations of Vienna’s system. They generally found that usage during peak hours of weekdays are quite different from that of weekends, and that differences in peak usage at stations might be associated with the kind of activities in the neighborhood. Beecham et al. [15] analyzed cycling trips by members of London’s bike-sharing system. They found that women tend to use public bikes at weekends and within London’s parks, while men tend to use public bikes for commuting. Moreover, women’s trips are highly spatially structured and mainly occur in areas with cycle routes and/or with slower traffic. Similar visual techniques were employed by Zhao et al. [16], who analyzed the cycling trip chains by gender and day of the week in Nanjing, China. They found that on weekdays, women tend to make multiple-circle trips and spend more time on cycling than men. Moreover, Zhou [17] investigated the spatial-temporal pattern of cycling trips of the Chicago bike-sharing system, and uncovered different travel patterns between weekdays and weekends as well as between customers and subscribers.

Secondly, to study the characteristics of the usage of bicycle-sharing systems, either for a single system or in a comparison of different systems. Jensen et al. [20] found that public bikes compete with the car in terms of speed in downtown Lyon by analyzing 11.6 million bicycle-sharing trips. Based on station data, Jäppinen et al. [19] indicated that integration of public bikes with traditional public transportation can promote sustainable daily mobility in Helsinki. Studies on London’s bicycle-sharing systems found that two strikes of the London subway led to an increase of the number and duration of public bike trips [18], and that easier access to the system can promote weekday commuting and weekend use [22]. Goodman and Cheshire [21] found that the introduction of casual access to London’s system encouraged more women to use the system, and the extension of the system to highly-deprived areas not only attracts new users but also increases local travel in such areas. O’Brien et al. [8] examined the usage of 38 global bicycle-sharing systems, and indicated that Asian systems have a lower compactness than European/Middle Eastern systems. They could also group Chinese systems together based on system attributes (e.g. system size, daily usage, etc.). Zhao et al. [35] compared 69 Chinese bike-sharing systems. Based on the effects of urban population, government expenditure, system size, and operation policy on daily use and daily use per bike, they suggested that the bike-member ratio could be less than 0.2 and that the adoption of personal credit and universal cards to access to systems influences the usage in a positive way.

Thirdly, to examine the impact of built environment factors and weather conditions on the demand at stations. In general, some studies found that population and job density, proximity to transit stations (metro and public bus stations) and bike lanes, and points of interests (retail shops, parks, restaurants, etc.) within the service area are positively associated with ridership at stations [23–32]. Moreover, station size and number of bike stations within the catchment area also have an impact on the bike-sharing demand at stations [25,26,28]. Severe weather conditions are associated with a negative impact on the system usage [33,34]. Finally, a small number of studies focuses on proposing a mathematical algorithm to deal with bike-sharing rebalancing problem [36–38].

Most of aforementioned studies, however, do not look at the dynamics of bike-sharing systems. Changes over time do not only occur in demand, but possibly also in the (type of) users. Do users and their demand change over time? This paper explores these questions in order to better understand the system and its future potential. It also investigates changes in usage over the years to identify which factors influence the system’s performance. This may provide useful insight for improving the location-allocation of current stations and for planning new stations. This study was conducted for a bicycle-sharing system in Zhongshan (China), using trip data from March 2012, March 2013, and March 2014. The system gradually expanded the number of stations equipped with parking slots between March 2012 and March 2013, and again between March 2013 and March 2014. To this end, we examined the changes in both users (UserID) and the system usage by comparing March 2012 with March 2013, and comparing March 2013 with March 2014. In this study, we consider the changes in the system as a whole as well as in the spatial distribution of demand before and after the system expansion.

## 3. Context and Methods

### 3.1. Study area

**Fig 1:**
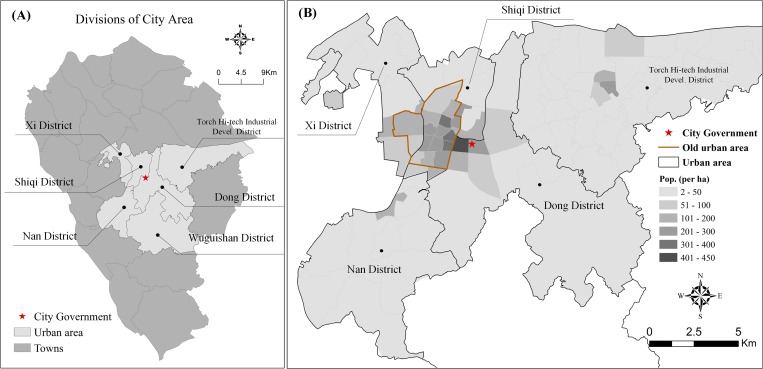
Study area. (A) Location and Divisions of study area; (B) Population density distributed in the study area. (Fig 1A and 1B were created by the author (Y. Zhang) based on the data, this is the original copyright.)

Zhongshan city is a medium-sized city that is located in the Guangdong province of China, and directly opposite Hong Kong. The city is a prefecture-level city (Fig 1A) whose government directly administers six districts corresponding to the urban area, and eighteen towns (in China, town is an administrative unit, into which counties and districts are divided). Among these, four districts—the Xi, Shiqi, Dong, and Nan districts—constitute the “major urban area” (Fig 1B), which covers an area of 170km^2^ and was home to a population of around 530,000 in 2013[39]. This major urban area can be characterized by a high population density and a concentration of residence, employment, shopping, entertainment, culture, and political power. In addition, the eastern and southern urban areas are the Torch Hi-tech Industrial Development district (90km^2^) and the Wuguishan district (113km^2^) respectively. The former is a national-level hi-tech industrial development zone with a population of 240,000 in 2013, and the latter is mainly intended for tourism and agriculture with a population of 48,000 in 2013.

According to travel statistics from Zhongshan transport planning department (this was done before running the bike-sharing program), non-motorized modes account for 46.3% of total trips, of which 24.3% are walking trips. The shares of motorcycle and private car trips are 39.8% and 8.5% respectively, whereas public bus trips only account for 4.2%. The average trip lengths in the major urban area are 0.8 km, 2.8 km, and 4.8 km for walking, cycling (bike and e-bike), and public bus trips respectively. In addition, 94.8% of all trips lasted less than 30 mins. In conclusion, non-motorized (walking and cycling) and motorcycle modes are the main travel modes in the “major urban area” while public transport is not very attractive to most residents.

### 3.2. Zhongshan’s bicycle-sharing system and data preparation

Zhongshan’s bicycle-sharing system was launched in October 2011 and is a 24/7 self-service system. Users can pick up and return public bikes at any station during the day, using a smart card that has a unique User-ID. Each user can apply for a smart card by registering as a member and depositing 200CNY. For each trip, the first hour is free, and any extra hours are charged at an incremental price (1CNY per hour), which is much cheaper than a trip by local public bus (2 CNY per trip).

**Fig 2:**
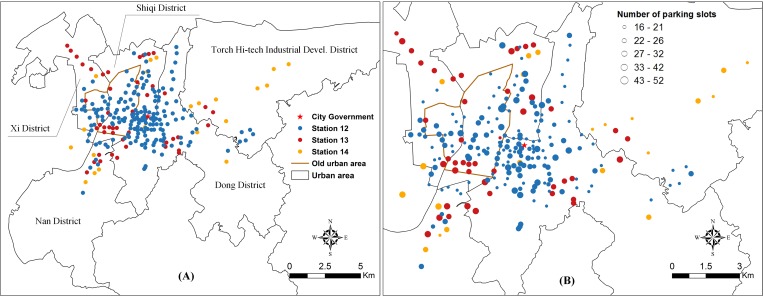
The spatial distribution of bike stations and capacities in the study area. (A) spatial distribution of stations that were built before and after the system expansion. (B) The number of parking slots at each of bike stations.

In the urban area, there were 180 bike stations equipped with 4530 parking slots in March 2012, increasing to 224 stations with 5959 parking slots in March 2013, and then further expanding to 245 stations equipped with 6547 parking slots in March 2014. The stations are shown in Fig 2, we use the label—“station12” for stations that were built before March 2012, label “station13” for stations that were built between March 2012 and March 2013, and label “station14” for stations built after March 2013. The average number of parking slots per station is 25, 32, 28 for “”station12”, “station13”, and “station14” respectively. Fig 2(A) shows how the system gradually expanded from the city center to the outskirts. It also shows that the density of stations is highest in the central area which has the highest population density and includes the city government. Fig 2(B) shows the size of the stations. It is worth mentioning that the majority of newly-built stations have quite a high capacity.

The data were collected from the Transport Department of the Urban Planning and Design Institute of Zhongshan (China). The provided trip data are from March 2012, March 2013, and March 2014, and include User-ID, pickup and return stations, and pickup time and return time. The duration of each trip is calculated by subtracting the pickup time from the return time. Based on data screening, we excluded two types of inaccurate records from the original trip database: (1) trips for which pickup or return information was missing; and (2) trips that lasted less than 1 minute, for which we assume no trip was actually made. As a result, we acquired data for 473,236 trips in March 2012, 453,846 trips in March 2013 and 398,305 trips in March 2014.

Weather conditions were considered as one of the potential factors that could have affected the bike use, but only extreme weather conditions (pouring rain or blistering heat) seem to really discourage cycling [40]. Zhongshan has a subtropical climate with an average temperature of 22°C and, in March, the weather is warm without strong winds. Rainfall was not extreme either and did not appear to have a significant influence on daily bike use. According to the statistical correlation between daily amount of rainfall (the whole day, as well as different time periods) and daily trips, the number of daily trips was not significantly (p<0.05) influenced by daily rainfall. We therefore did not consider weather conditions in the further analysis.

### 3.3. Methods

This study aims to explore how the usage of the system changes following system expansion. To this end, we performed both statistical and spatial analyses to examine the changes in both *users* and *system usage* between March 2012 and March 2013, and between March 2013 and March 2014. The analyses were carried out using SPSS and ArcGIS. We separate travel on weekdays from weekends and also distinguish between morning peak hours and evening peak hours.

Comparing “User-IDs” before and after the system expansion, users are divided into three groups: (1) *former users* who used the system before the system expansion but not at all after the system expansion; (2) *steady users* who used the system both before and after the system expansion; and (3) *new users* who started to use public bikes only after the system expansion.

The system usage was investigated by: (1) the aggregate use of the system and (2) the spatial distribution of both users’ demand and the ratio of demand to supply (D/S). We examined the system usage for both all users and per user group. The aggregate use of the system is based on daily usage (distinguishing weekdays and weekends) and hourly usage (distinguishing morning peak hours and evening peak hours). The definition of morning peak hours and evening peak hours is based on the number of trips generated over the hour of day. Morning peak hours are 7:00–9:00 on weekdays and 8:00–9:00 on weekends, and evening peak hours are 17:00–19:00 on both weekdays and weekends. Daily and hourly usage were described by the usage metrics which mainly include the average number of users, average number of trips, average number of trips per user, average number of demands per station (distinguishing between “old” stations and newly-built stations), average trip length, and average trip duration. The number of trips corresponds with the demand for bikes, as one trip means a user picks up a bike from a station and returns the bike to another or the same station. The demand at each station was calculated by the sum of departure trips (i.e. picking up bikes) and arrival trips (i.e. returning bikes) at the station, as the number of pick-ups is comparable to the number of returns at each station (see S1 Fig).We decided to use the “Median” to calculate the “average” value of aforementioned usage metrics, which can mitigate the impact of some outliers (e.g. sharp decrease) on the measure of daily use.

The spatially oriented approach provides operators and researchers with a better understanding of usage and user patterns [41]. The spatial distribution of both demand and D/S was used to uncover the trend in distribution of bike-sharing use across the urban area. Moreover, the D/S can be an indication of the relationship between users’ demand and system’s supply. The users’ demand refers to the average number of trips generated by a group of users, which is a metric of the aggregate use of the system. The system’s supply refers to the number of parking slots, which was not a constant and increased after the system expansion.

**Fig 3:**
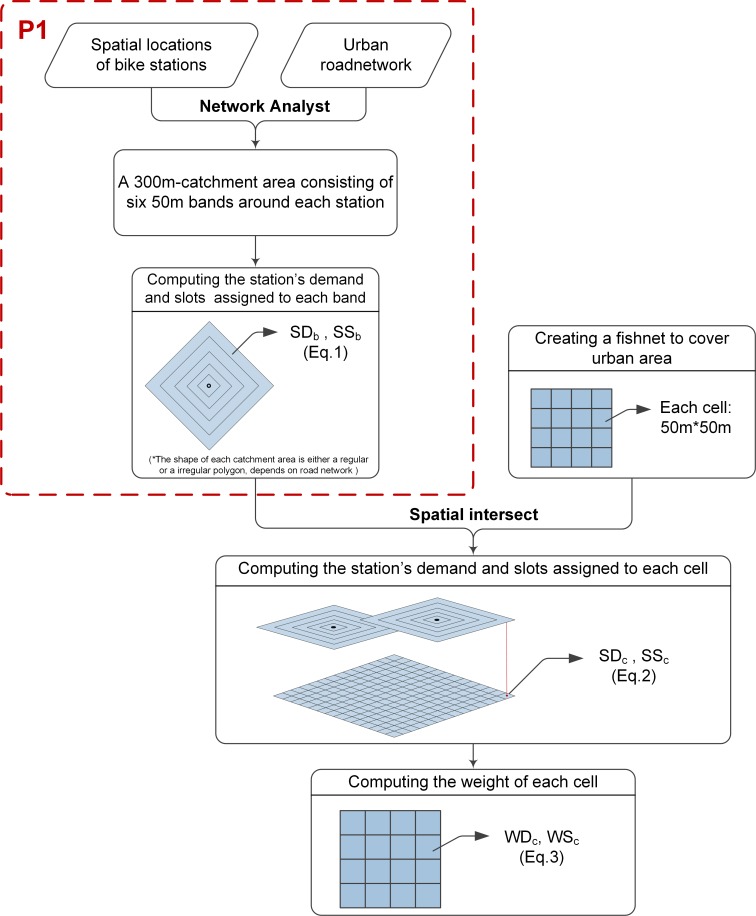
A diagram of computing the weight of each cell.

The spatial distribution was visualized by a spatial fishnet that divided the urban area into a bunch of grid cells. The spatial fishnet was created in ArcGIS, with each cell having a size of 50 by 50 meters. Fig 3 shows how we computed the weight of each cell, which determines the relative importance of each cell, and lays a foundation for smoothing the overall users’ demand and system’s supply over grid cells.

The Eqs 1and 2 show how we smoothed the demand (left column) and supply (right column) at stations (discrete locations) over the cells. We first created a catchment area (300m radius) consisting of six 50-m concentric bands, around each bike station (see P1 in Fig 3). The size of the catchment area was chosen such that it is approximately equal to 344 m average distance between neighboring stations. This catchment area also corresponds with a suitable walking distance. The catchment areas were generated based on network distances (the shape of each catchment area is regular or irregular polygon depending on the road network), which are considered for walking to transit facilities. We then redistributed the station’s demand and supply (i.e. slots) to each band based on distance decay (Eq 1), that is, *SD*_*b*_ and *SS*_*b*_. This decay actually represents the distribution of the users’ actual origins or destinations around each station. In other words, users are more likely to use stations when they are very nearby, but they can still use a station if they have to walk some distance. Afterwards we carried out a spatial analysis to intersect catchment area (bands) with spatial fishnet (grid cells) to distribute the bands’ demand (*SD*_*b*_) and supply (*SS*_*b*_) to each grid cell (Eq 2). Further, Eq 3 shows how we assigned a weight of demand (*WD*_*C*_) and a weight of supply (*WS*_*C*_) to each grid cell and the sum of each cell’s *WD*_*C*_ (and *WS*_*C*_) is 1.

The Eq 4, *D*_*c*_ and *S*_*c*_, represent the number of users’demand and system’s supply respectively, which is given to each cell. For example, the spatial distribution of demand and the spatial distribution of D/S (the ratio of *D*_*c*_ to *S*_*c*_) over the cells, are shown in figures of section 4.2.1.

In the results section, we describe the trends in spatial distribution of users’ demand and D/S, and employ Hot Spot analysis (spatial statistics in ArcGIS) to identify statistically significant hot spots and cold spots for users’ demand using Getis-Ord Gi* statistic. This may uncover whether there are significant differences in the spatial distribution of users’ demands following the system expansion. We also examine the differences in number of demands between user groups over the grid cells. However, there is a considerable difference in total demand between the different groups. To take this difference into account, we normalized the demand of user groups. As an example, Eq 5 shows how we calculated the difference in spatial demand (i.e. *D*_*c*_) between U12 and U13, which is users in 2012 and users in 2013. In Eq 5, the factor *α* is the ratio of overall demand between U12 and U13. The function of *ND*_*c*(*U*12)_ is used to normalize the cells’ demand of U12, and consequently the sum of *ND*_*c*(*U*12)_ is equal to the sum of *D*_*c* (*U*13)_. The function of *ND*_(13*vs*12)_ calculates the normalized difference in each cell’s demand between U12 and U13. As a result, the values of *ND*_(13*vs*12)_ of all cells are normally distributed with a mean 0 and a sum 0; we therefore use standard deviation as a unit to visualize the difference in demand over grid cells, as shown in figures of section 4.2.2.

$${SD}_{b}=D_{i,j}=D_{i}^{2}/(d_{j}*\sum_{j}D_{i}/d_{j})\;{SS}_{b}=S_{i,j}=S_{i}^{2}/(d_{j}*\sum_{j}S_{i}/d_{j})$$ Eq. 1

$${SD}_{C}=\sum_{b=1}^{n}{SD}_{b}*{ap}_{b}\;{SS}_{c}=\sum_{b=1}^{n}{SS}_{b}*{ap}_{b}$$ Eq. 2

$${WD}_{C}={SD}_{c}/\sum_{c}^{n}{SD}_{c}\;{WS}_{C}={SS}_{c}/\sum_{c}^{n}{SS}_{c}$$ Eq. 3

$$D_{c}={WD}_{c}*D_{user}\;S_{c}={WD}_{s}*S_{system}$$ Eq. 4

$${ND}_{\left(13vs12\right)}=D_{c\;(U13)}-{ND}_{c(U12)}\;{ND}_{c(U12)}=D_{c(U12)}/\alpha \;\alpha =D_{U12}/D_{U13}$$ Eq. 5

Where:

- *b* is the ID of each band (*b* = 1,…*n*), and *i* is the ID of each bike station;
- *d*_*j*_ is the distance of the band, *d*_*j*_ = 50m,100m,150m,200m,250m,300m, in which *j* = 1,2,3…6 respectively;
- *D*_*i*_ is the number of demands at station *i*; *S*_*i*_ is the number of parking slots at station *i*;
- *ap*_*b*_ is the area proportion of each cell that spatially overlaid with distance band *b*;
- *D*_*user*_ is the average daily trips generated by a user group;
- *S*_*system*_ indicates the amount of parking slots.

## 4. Results and Discussions

In this section, we present and discuss the results of two aspects. Section 4.1 presents the aggregate use of the system by different user groups before and after the system expansion. Section 4.2 presents the trends and the changes in spatial demand by users between before and after the system expansion. Travel on weekdays was analyzed separately from travel on weekends and we also distinguish between morning peak hours and evening peak hours.

### 4.1. Aggregate use of the system before and after system expansion

Table 1 describes the aggregate daily and hourly use of the system by all users, on weekdays and weekends of March 2012, March 2013, and March 2014. It reveals there is an overall decrease in daily use between 2012 and 2014, despite the expansion of the system. This decrease is most distinct in weekends. Not only does the number of users decrease, the average number of trips per user is also declining. The expansion of the system has resulted in extra usage at new stations (shown in the rows “daily demand per station13” and “daily demand per station14” for the station added in 2013 and 2014 respectively). In 2013, this was quite a substantial part of the total, actually resulting in an overall increase in usage for workdays between 2012 and 2013. However, after the second expansion the number of new trips per added station decreased (81 for stations added in 2013 and only 25 for stations added in 2014). This shows that stations added in 2014 have less demand than stations added in 2013, which can be attributed to the fact that the majority of “station14” is on the outskirts. The fact that newly-built stations have not led to an overall increase in demand can be attributed to a significant decline in usage at the original stations (from 182 per station 12 in 2012 to 139 per station 12 in 2014). Partly, this can be explained by the fact that new stations might compete with older stations, but the rate of decline is somewhat surprising. To provide a better interpretation of this result, we need to consider different user types, which will be done in Tables 2 and 3..

**Table 1:** The aggregate use of the system by all users in March 2012, March2013, and March 2014.

Usage metrics	Weekdays			Weekend		
	2012 (22 days)	2013 (21 days)	2014 (21 days)	2012 (9 days)	2013 (10 days)	2014 (10days)
Daily users^a^	9075	9429	8374	8803	8328	6789
Daily trips	16292	16481	14562	15570	14703	11398
Daily trips/user	1.79	1.75	1.71	1.77	1.74	1.68
Daily demand per station12	182	167	139	174	147	109
Daily demand per station13	-	71	81	-	69	65
Daily demand per station14	-	-	25	-	-	23
Hourly trips (MP)^b^	1460.75	1664.5	1523	1080	1165	1106
Hourly demand per station 12 (MP)	13.5	13.83	11.50	9.67	8.1	7.40
Hourly demand per station 13 (MP)	-	6.67	6.93	-	3.7	4.30
Hourly demand per station 14 (MP)	-	-	1.43	-	-	1.30
Hourly trips (EP)^c^	1886.5	1878	1628	1552	1362.5	1122.25
Hourly demand per station 12 (EP)	15.94	13.71	11.93	12.36	9.15	7.15
Hourly trips per station 13 (EP)	-	5.76	7.74	-	4.05	4.50
Hourly trips per station 14 (EP)	-	-	1.59	-	-	1.15
Trip length (m)	1356	1334	1346	1334	1299	1321
Travel time (minutes)	11	10	10	12	11	11

^a^ One user represents a User-ID that belongs to a specific person.

^b^ “MP” is the abbreviation for “morning peak hours”.

^c^ “ËP” is the abbreviation for “evening peak hours”.

**Table 2:** The aggregate use of the system by steady users, former users, and new users on weekdays.

Usage metrics (Median)		Mar 12 vs Mar 13		Mar 13 vs Mar 14	
		2012	2013	2013	2014
Steady users	Daily users	4864	4127	4888	4044
	Daily trips	8842	7024	8793	6756
	Daily trips/user	1.8	1.69	1.78	1.66
	Daily demand per station12	99	73	89	66
	Daily demand per station13	-	24	36	36
	Daily demand per station14	-	-	-	12
	Hourly trips (MP)	838.25	743.5	939	750
	Hourly demand per station 12 (MP)	7.99	6.36	7.45	5.83
	Hourly demand per station 13 (MP)	-	2.18	3.62	3.5
	Hourly demand per station 14 (MP)	-	-	-	0.48
	Hourly trips (EP)	1010.25	797	992	751
	Hourly demand per station 12 (EP)	9.03	6.19	7.36	5.69
	Hourly trips per station 13 (EP)	-	2.10	3.12	2.64
	Hourly trips per station 14 (EP)	-	-	-	0.33
	Trip length (m)	1337	1268	1337	1314
	Travel time (minutes)	10	10	10	10
Former Users Vs New Users	Daily users	4193	5252	3939	4311
	Daily trips	7419	9401	6786	7749
	Daily trips/user	1.76	1.78	1.70	1.75
	Daily demand per station12	83	93	68	74
	Daily demand per station13	-	46	30	46
	Daily demand per station14	-	-	-	17
	Hourly trips (MP)	617.5	950	653	796
	Hourly demand per station 12 (MP)	5.82	7.05	5.06	5.57
	Hourly demand per station 13 (MP)	-	3.76	2.40	3.38
	Hourly demand per station 14 (MP)	-	-	-	1.36
	Hourly trips (EP)	865.5	1085.5	775	883
	Hourly demand per station 12 (EP)	7.15	7.33	5.48	6.31
	Hourly trips per station 13 (EP)	-	3.93	2.19	4.12
	Hourly trips per station 14 (EP)	-	-	-	1.43
	Trip length (m)	1375	1379	1337	1384
	Travel time (minutes)	11	11	10	10

**Table 3:** The aggregate use of the system by steady users, former users, and new users on weekends.

Usage metrics (Median)		Mar 12 vs Mar 13		Mar 13 vs Mar 14	
		2012	2013	2013	2014
Steady users	Daily users	4555	3449	4206	3228
	Daily trips	8127	5961	7569	5304
	Daily trips/user	1.79	1.69	1.77	1.64
	Daily demand per station12	91	61	76	52
	Daily demand per station13	-	23	35	29
	Daily demand per station14	-	-	-	9
	Hourly trips (MP)	895	492.5	636	531.5
	Hourly demand per station 12 (MP)	5.11	3.5	4.4	3.5
	Hourly demand per station 13 (MP)	-	1	1.8	2.05
	Hourly demand per station 14 (MP)	-	-	-	0.4
	Hourly trips (EP)	801.5	530.5	690	473
	Hourly demand per station 12 (EP)	6.56	3.75	4.6	3.2
	Hourly demand per station 13 (EP)	-	1.55	2.25	2.0
	Hourly demand per station 14 (EP)	-	-	-	0.18
	Trip length (m)	1318	1238	1295	1266
	Travel time (minutes)	11	10	11	10
Former Users Vs New Users	Daily users	4125	4823	3631	3545
	Daily trips	7189	8591	6295	6073
	Daily trips/user	1.74	1.78	1.71	1.71
	Daily demand per station12	80	85	63	58
	Daily demand per station13	-	46	29	36
	Daily demand per station14	-	-	-	15
	Hourly trips (MP)	484	671.5	468.5	571.5
	Hourly demand per station 12 (MP)	3.89	4.4	3.1	4.2
	Hourly demand per station 13 (MP)	-	2.3	1.3	2.3
	Hourly demand per station 14 (MP)	-	-	-	1.2
	Hourly trips (EP)	746	830	598.5	639.5
	Hourly demand per station 12 (EP)	5.56	5.1	3.85	3.9
	Hourly trips per station 13 (EP)	-	3	1.7	2.5
	Hourly trips per station 14 (EP)	-	-	-	1.07
	Trip length (m)	1350	1337	1316	1362
	Travel time (minutes)	12	11	11	11

**Fig 4:**
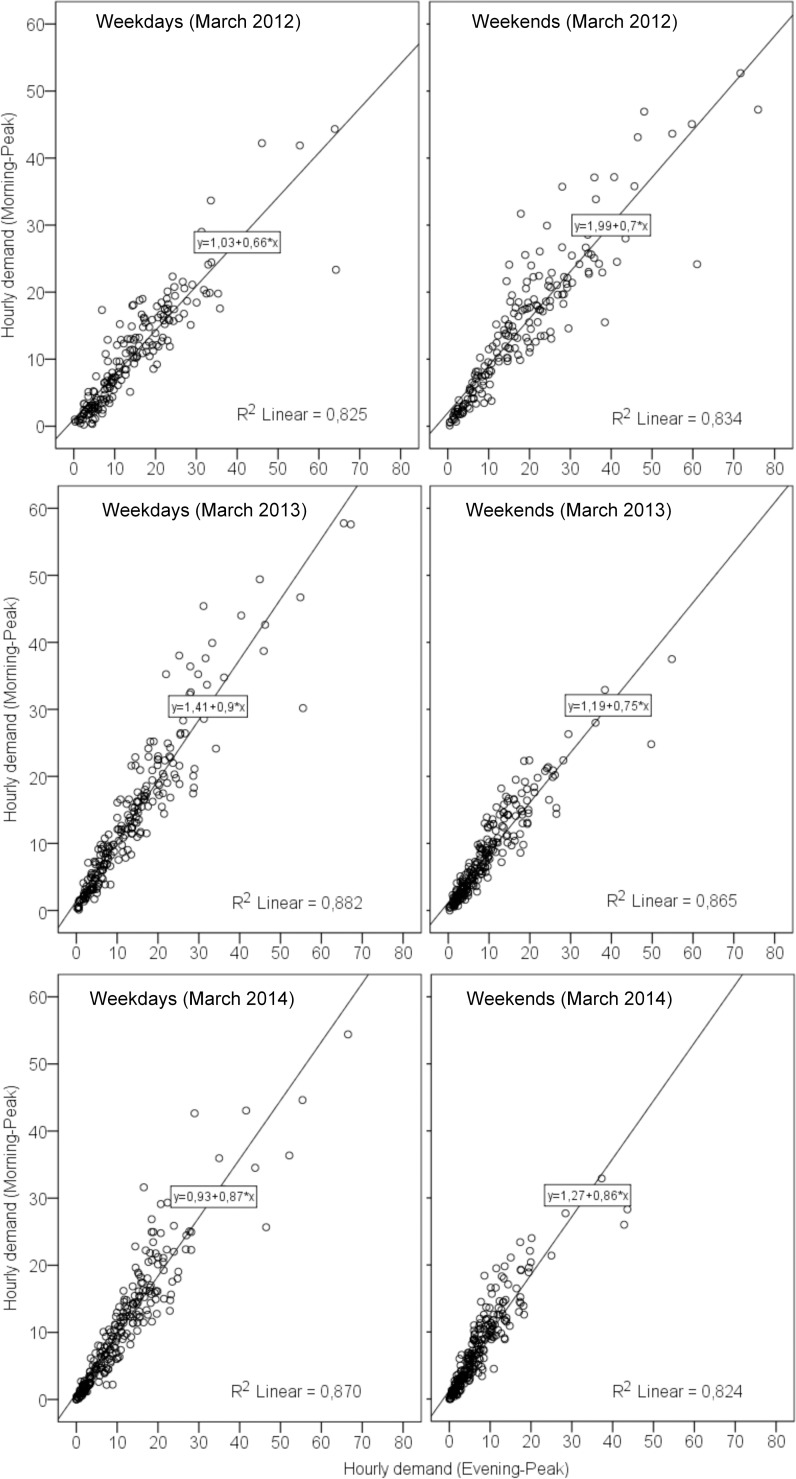
Comparisons of hourly demand during morning peak and evening peak hours at stations. (1) the points represent each of bike stations. (2)Y axis represents the average number of hourly demand during morning peak hours, and X axis represents the average number of hourly demand during evening peak hours.

In addition, the change in hourly usage during morning peak and evening peak hours is comparable with the change of daily usage following the system expansion. Regarding the comparisons of hourly usage during morning peak hours and evening peak hours, the users’ demand (the average number of hourly trips) during evening peak hours is slightly larger than during morning peak hours. This might be attributed to more people use the system (or users generated more trips) during evening peak hours, because people have more leisure activities (or spare time) in the evening (after work) than in the morning. When we look at the demand at stations, there is no considerable difference in hourly demand per station between morning peak hours and evening peak hours, especially after the system expansion. Moreover, Fig 4 describes the comparison of the number of hourly demands at each station between morning peak hours (Y axis) and evening peak hours (X axis). This indicates that the number of hourly demands at each station during morning peak is comparable with that during evening peak, especially in March 2013 and March 2014. Fig 4 also indicates that bike stations that have high demand during morning peak hours also generate a high demand during evening peak hours. This implies that the spatial distribution of demand during morning peak hours is similar to that during evening peak hours. Finally, there is no significant difference in trip characteristics between, before and after the system expansion: generally the average trip length and average trip duration are both quite short.

Users are divided into three groups–*former users*, *steady users*, and *new users*–based on the comparison of User-IDs between, before and after the system expansion. Table 2 and Table 3 describe the division of user groups and the aggregate use of the system by each user-group on weekdays and weekends respectively. For each user group, the number of users on weekdays is higher than that on weekends, demonstrating that some users only used the system on weekdays.

Table 2 describes the aggregate daily and hourly use of the system by each user group, on weekdays and weekends of March 2012, March 2013, and March 2014. It shows that there is a great variation in users. About only half of the users are steady users (when comparing between successive years), while the rest are former or new users. This indicates that the system is quite dynamic and has not (yet) found some form of equilibrium. The system is also quite new and still expanding. Interestingly, there are more new users than former users. They use the system more frequently and also make more trips than former users. However, as we have seen, the overall demand has declined over time. This can be attributed to the steady users. These users have used the system less frequently over time (resulting in a decrease of the number of users per day), and also made fewer trips (resulting in a decrease in the number of trips per user per day). These trends are both visible for workdays and weekends. It is not clear why there is a decline in usage among steady users, especially in the light of an expanding system. To provide better interpretation of these results, we investigated the spatial distribution of demand before and after system expansion, which will be presented in the next subsection.

### 4.2. Spatial distribution of demand before and after the system expansion

#### 4.2.1. Trends in the spatial distribution of demand and D/S

In this subsection, we explore the spatial distribution of demand and demand over supply (D/S) before and after the system expansion. As mentioned before, the spatial distribution of hourly demand at stations during morning peak hours is comparable with that during evening peak hours (i.e. high and low demand at stations), we therefore only use daily usage to describe and compare the spatial distribution of demand and D/S between before and after system the system expansion.

**Fig 5:**
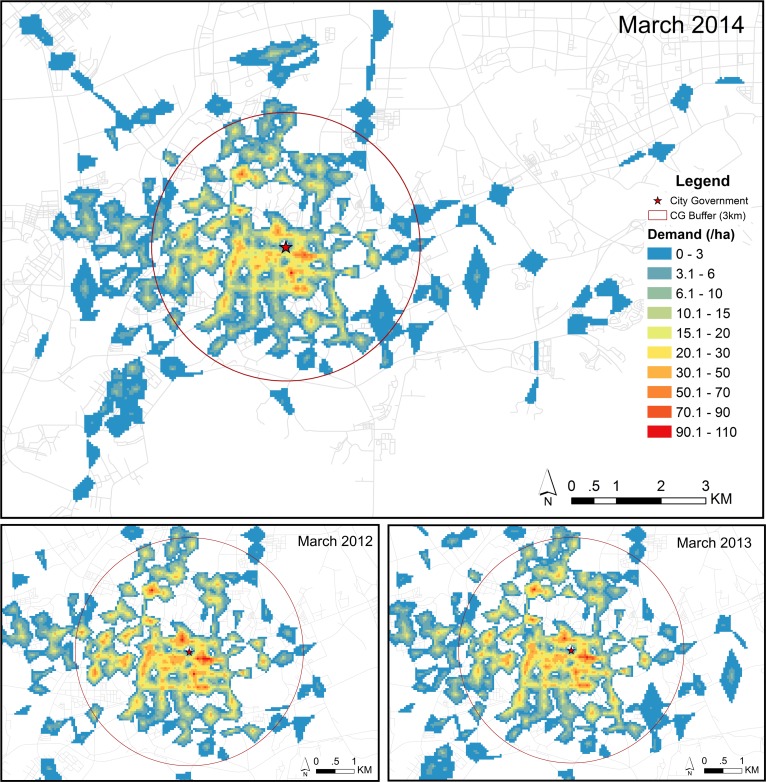
The spatial distribution of demand by all users on weekdays. Demand represents the number of trips per day. The upper panel represents the result of March 2014. The lower panel represents the results of March 2012 (left) and March 2013 (right).

**Fig 6:**
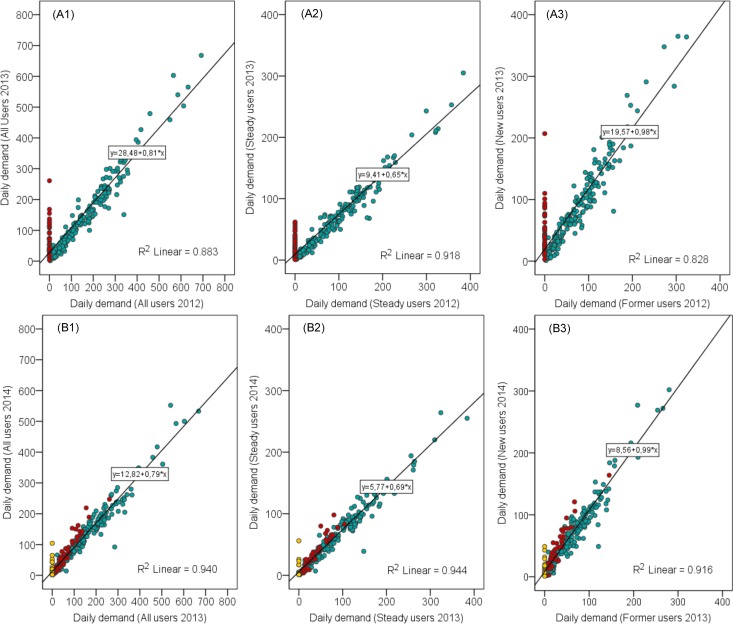
Comparisons of daily demand by one group and another group at stations (weekdays). (1) The points represent each of bike stations. (2) Blue, red, and yellow symbols denote “stations 12”, “stations 13”, and “stations 14” respectively. (3) Figures A describe the user groups of Mar 2012 versus Mar 2013, and figures B describe user groups of Mar 2013 versus Mar 2014.

**Fig 7:**
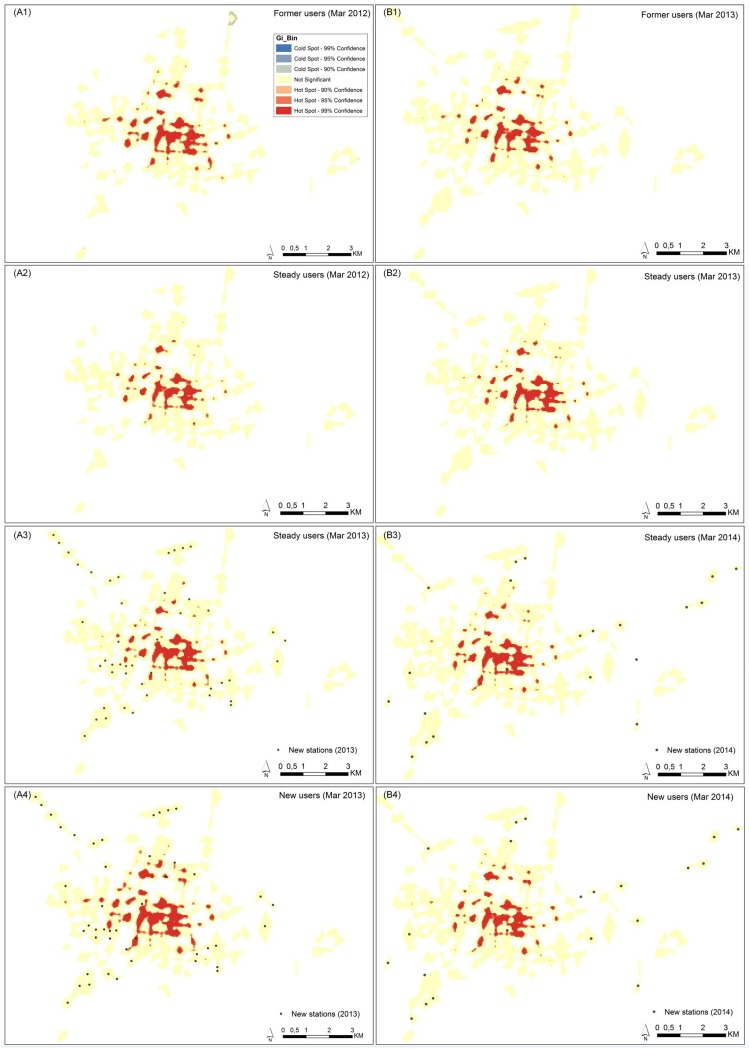
Getis-Ord Gi* statistic of the spatial distribution of demand by each user group (weekdays). Figures A describe the user groups of Mar 2012 versus Mar 2013, and figures B describe user groups of Mar 2013 versus Mar 2014.

Fig 5 shows the spatial distribution of demand (i.e. trips/day) by all users on weekdays in March 2012, March 2013, and March 2014. As expected, the demand is the highest in the central part of the city and drops towards the outskirts. Similar trends can also be observed for each user group–former users, steady users, and new users. Fig 6 displays the comparison of the number of daily demands at each station between different user groups on weekdays in March 2012, March 2013, and March 2014. This indicates that stations, which generate a high (and low) demand by one user group, also generate a high demand (and low) demand by another user group. It implies that there is no considerable difference in spatial distribution of demand between different user groups. Moreover, Fig 7 shows the spatial distribution of statistically hot spots of demand (i.e. trips/day) by each user group on weekdays in March 2012, March 2013, and March 2014. This further confirms that there are no substantial differences in the pattern of spatial distribution of demand between different user groups. The statistically significant hot spots are generally the same among different user groups (i.e. following the system expansion). This suggests that the spatial distribution of demand before the system expansion is comparable with that after the system expansion, as well as between different user groups. In all cases, high-demand areas concentrate in the center, whereas the low-demand areas are on the outskirts. This might be attributed to the fact that the central area has the highest density of population, bike stations, and mixed land use patterns.

**Fig 8:**
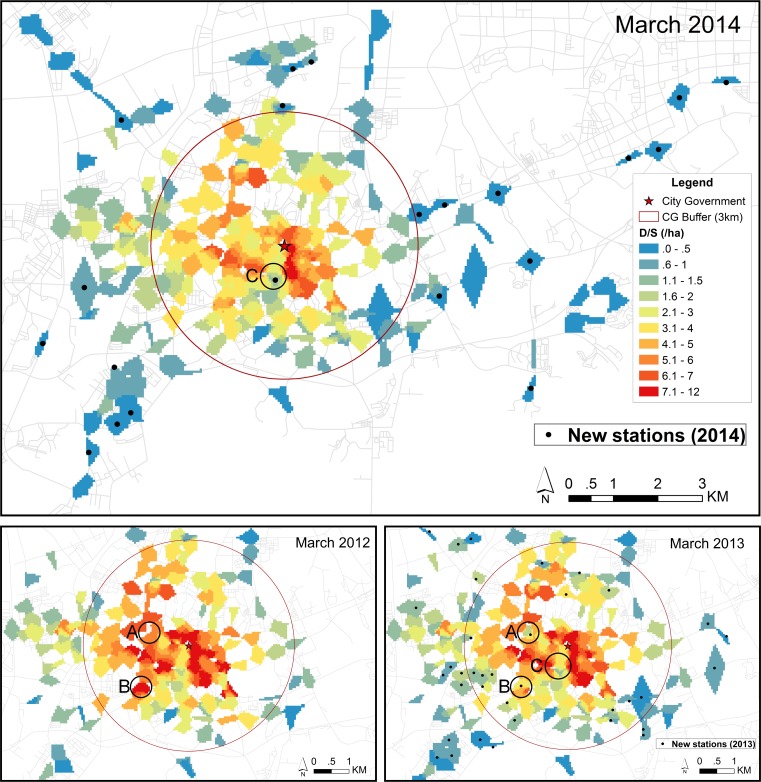
The spatial distribution of D/S by all users on weekdays. The D/S represents the ratio of trips/day to the number of parking slots. The upper panel represents the result of March 2014. The lower panel represents the results of March 2012 (left) and March 2013 (right).

Fig 8 shows the spatial distribution of D/S (i.e. the ratio of trips/day to slots) on weekdays in March 2012, March 2013, and March 2014. Not surprisingly, it somewhat follows the trend in demand with high D/S in the central area and low D/S on the outskirts. The figure however shows an overall decrease in D/S following the system expansion, especially in the central area, the color changes from upper class to lower class (such as from 7.1–12 to 6.1–7). This might be attributed to the overall decrease in demand by all users following the system expansion. In addition, some of areas, which showed a high D/S before system expansion, decreased after building a new station nearby, such as marked in the areas A, B, and C shown in in Fig 8. The overall demand in those areas has increased after system expansion, due to the demand for new stations. This suggests that new stations might compete with nearby older stations, resulting in mitigating the excess demand (finding available bikes or empty slots) in areas that had a high D/S before the system expansion.

**Fig 9:**
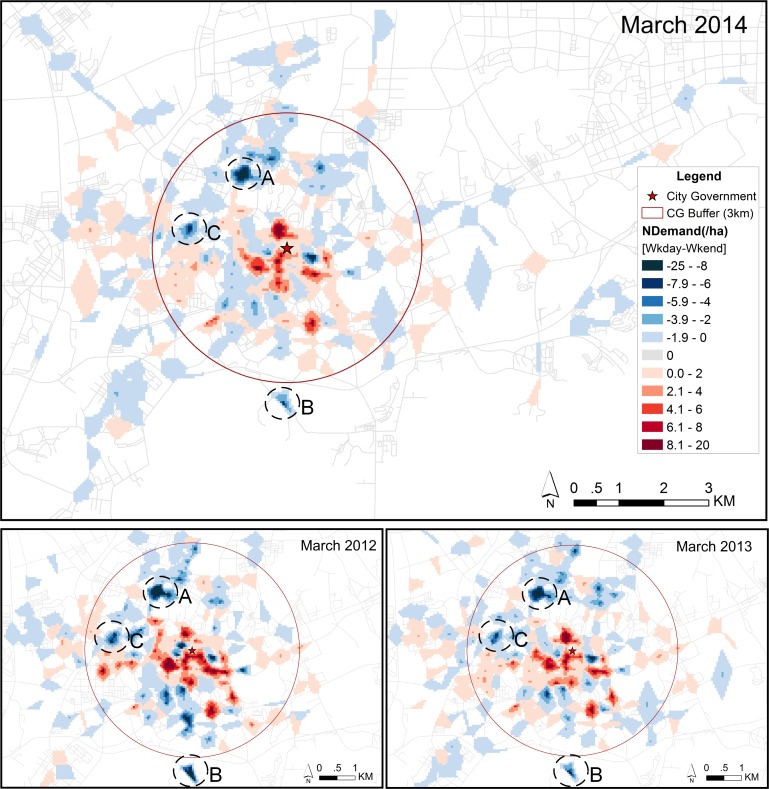
Differences in spatial demand by all users between weekdays and weekends. The “NDemand” represents the difference in normalized demand between weekdays and weekends. The upper panel represents the result of March 2014. The lower panel represents the results of March 2012 (left) and March 2013 (right).

In the previous subsection, we found that the overall use of the system has decreased more on the weekends than on weekdays. Therefore, we examined the spatial difference in demand between weekdays and weekends. The results are shown in Fig 9. Although the overall spatial distribution looks quite similar for weekends and weekdays, Fig 9 shows there are differences in the number of demands. The red areas show a relatively higher demand on weekdays, while the blue areas show relatively higher demand on weekends. The figure shows that these areas are more or less the same in the three years.

This result suggests that differences between weekdays and weekends are not related to the expansion of system, but probably to the surrounding built environment. Blue areas are mainly occupied by shopping malls or parks, distributed far from the city center, marked as areas A, B, and C in Fig 9. The significant red areas are mainly located in the city center, with relatively many offices and residential communities. This implies that commuting is more dominant on weekdays, and shopping and recreation are more important purposes in the weekends. These results suggest that the demand in an area is influenced by the nearby dominant land use type.

#### 4.2.2. Differences in spatial demand by user groups

**Fig 10:**
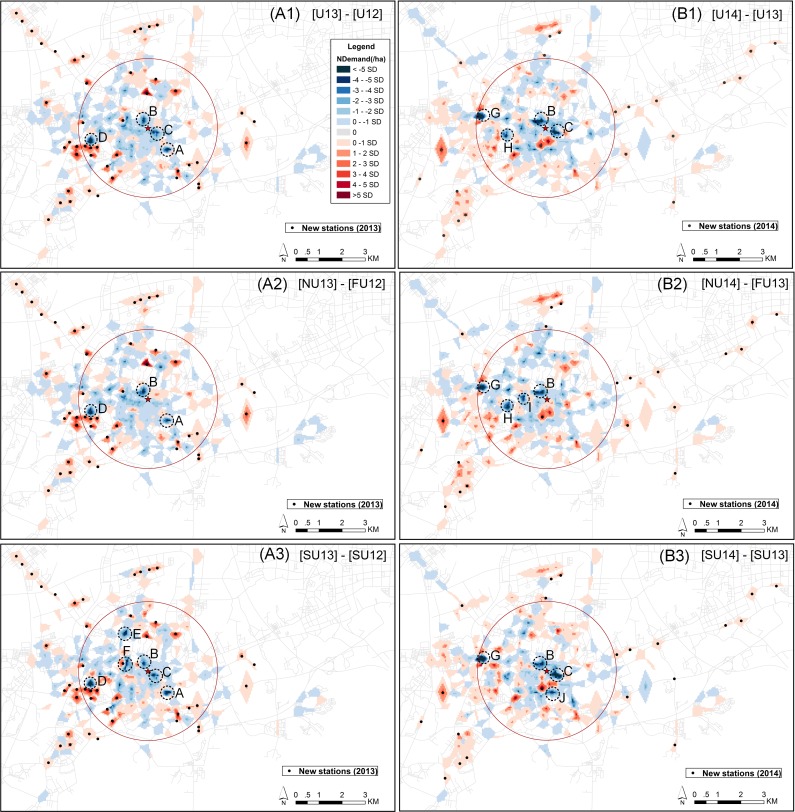
Differences in spatial demand between user groups of before and after the system expansion (weekdays). The “NDemand” represents the difference in normalized demand between user groups. The left panel represents the comparison between March 2012 and March 2013, and right panel represents the comparison between March 2013 and March 2014.

**Fig 11:**
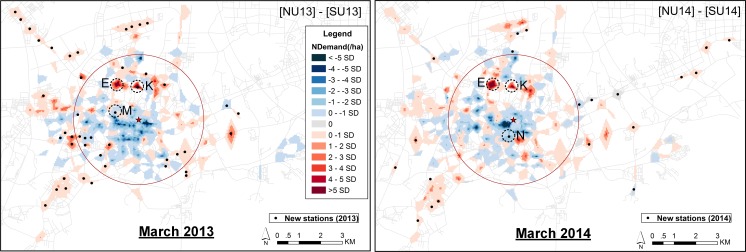
Differences in spatial demand between new users and steady users after system expansion (weekdays). The “NDemand” represents the difference in normalized demand between new users and steady users. The left panel represents the comparison between new users and steady users in March 2013. The right panel represents the comparison between new users and steady users in March 2014.

In this section, we focus on two aspects. The differences in spatial demand between the three years are shown in Fig 10. This is done for all users (upper panel), new versus former users (center panel), and for steady users (lower panel). The differences in spatial demand between new users and steady users after the system expansion are shown in Fig 11.

The red areas in Fig 10 show that the demand after system expansion is higher than before the system expansion (higher in 2013 than in 2012, left panel; and higher in 2014 than 2013, right panel). The blue areas are areas in which demand has decreased. Note that the most significant increases (illustrated by deep red colors) are in areas with newly-built stations. According to Fig 10, decreases in demand are mainly observed in central areas. We highlight areas with the most significant decrease (i.e. more than 4 times the standard deviations below the average normalized difference of 0) in each figure. These areas are not necessarily the same when comparing the second expansion with the first one, or when comparing new users (vs. former users) with steady users.

Area A is a specific case. The strong decrease in 2013 (compared to 2012) is due to the removal of a station. The other stations show real decreases in demand. Area B, C, and E show a significant decrease throughout all years, and area B for all groups but area C and E for steady users. However, these areas are constantly high-demand areas throughout three years, area B and E are occupied by a shopping mall and area C is occupied by a mix of offices and residential communities. The continuous decline of demand in these areas might be attributed to the negative performance of the system, such as the quality of bikes is not as good as the beginning, and unavailability of bikes or parking slots.

Additionally, decreases in other areas area only significant in one of the two expansions. However, we observe a decrease in all cases. It should be noted that demand–by both new users (vs. former users) and steady users–has decreased in areas where (many) new stations were added nearby. This is in particular the case for area D, F and J that are occupied by the mix of offices and residential communities. The case for area G that is a commercial area consisting of hotels, shopping malls, and entertainment venues, where a new station was added nearby in the first expansion, decreased demand in 2013 (left panel) and shows a significant decrease of demand after the second expansion (right panel). However, the demand in newly-built areas increased after second expansion (right panel). This implies that there might be competition between nearby stations, where newly-built stations are more attractive than the older stations. Two specific areas H and I–that are occupied by residential communities (area H) and the mix of colleges, residential communities and a park (area I)–only show a significant decrease in demand by new users (vs. former users) after system expansion. This might be due to fewer new users have demand for stations in these areas, such as people living, studying or working in this location.

Fig 11 shows the differences in spatial demand between new users and steady users after system expansion, i.e. comparing new users with steady users in March 2013 (left panel), and in March 2014 (right panel). March 2013 and March 2014 show similar patterns. In the blue areas the demand is relatively high for steady users, while in the red areas the demand is relatively high for new users. For the majority of areas, the difference in demand between steady users and new users is not very high. Fig 11 illustrates that steady users show the higher demand for both old stations and new stations that are located in the city center, such as the newly-built areas M and N. This can be attributed to the fact that the activities of steady users were mainly concentrated in the central area before the system expansion; users have more desires for newly-built stations in this area rather than the new stations that are far away. New users generated a higher demand at the majority of new stations as well as at some old stations nearby shopping malls–as areas E and K. This implies that adding new stations in the areas where demand or density of stations is high, both new users and original users can be attracted. On the other hand, adding new stations in areas further away from the city center, with a lower density of stations, is mainly useful for new users rather than steady users. In general, expanding the original system not only extends the original users’ ability to reach new areas but also attracts new users to use bike-sharing systems.

## 5. Conclusions

This study has investigated how the system usage has changed over the years and how the system expansion affects the usage of the system. It was performed to evaluate Zhongshan’s bicycle-sharing system, using trip data from March 2012, March 2013, and March 2014. The system gradually expanded the number of stations equipped with parking slots from March 2012 to March 2013 and then again from March 2013 to March 2014. We conducted both a statistical and a spatial analysis to examine the changes in both users and system usage between before and after the system expansion, namely March 2012 versus March 2013 as well as March 2013 versus March 2014. The system usage was measured by: (1) the aggregate use of the system; and (2) the spatial distribution of users’ demands and the ratio of demand to supply (D/S). In addition, travel on weekdays was analyzed separately from travel on weekends.

There has been a great variation in the number of users over the years, with only 45%-46% of all users–steady users–continuing to use the system after the system expansion. Many users–former users–stopped using the system, and many new users started to use the system after the system expansion. Moreover, there are overall decreases in the system usage by all users after the system expansion compared to before the system expansion, due to the overall decreases in the system usage by steady users after the system expansion, although new users used the system more frequently than former users.

There is no significant difference of the trend in spatial distribution of both demand and D/S between, before and after the system expansion. The high-demand areas concentrate in the center and are occupied by old stations, and the low-demand areas are on the outskirts. This is attributed to the fact that the center area has the highest density of population, bike stations, and mixed land use patterns. However, there were decreases in demand in most high-demand areas over the years, due to a reduced demand by both steady users and new users (versus former users). This implies that stations in these high-demand areas did not work well after the system expansion compared to before the system expansion, which is not attributed to the system expansion, but might be caused by the fact that the novelty was gone for some steady users or the negative performance of the system, such as the quality of bikes not being as good as in the beginning, and unavailability of bikes or parking slots.

In some areas which are occupied by both old and new stations after the system expansion, less demand by both new users and steady users was generated at these old stations after the system expansion, compared with the demand by former users and steady users before the system expansion. Moreover, the spatial distribution of D/S reveals that these areas showed a high D/S before the system expansion, but decreased the D/S after building a new station. This suggests that nearby stations might be competing with each other, and building new stations in former high D/S areas can contribute to easing the excess demand in these areas. In addition, the difference in demand over the urban area between weekdays and weekends reveals that users might cycle mainly for commuting on weekdays, but for shopping and recreation on weekends.

In general, expanding the original system not only extended the original users’ ability to reach new areas but also attracted new users to use the bike-sharing system. Adding new stations in the areas where demand or density of stations is high can attract both new users and original users. On the other hand, adding new stations in areas further away from the city center with a lower density of stations is mainly useful for new users rather than steady users.

With the development of a bike-sharing system, to improve the system and make it more sustainable rather than a short-lived project, this study is aligned with a tendency for operators and researchers to investigate the system usage and travel behaviors of bike-sharing users by the trip data that discloses more information than the station-based data. That was also the motivation for us to conduct this study. To be sure, this study is not without limitation. Due to the data limitation, we only compared the one-moth system usage between three years. It would be better to collect and analyze the trip data over the long term, which may make the results of analysis more conclusive. This is an avenue for future work.

For further expansion of bike-sharing systems, we suggest that it would be better to first investigate the spatial patterns of users’ demands and system’s supply to uncover the high and low level of demand as well as the ratio of demand to supply across the urban area. Next, we suggest building new stations in the area that has an excessive ratio of demand to supply rather than expand the system to new areas unless there is a clear necessity for serving new areas. Building new stations in the areas with high ratio of demand to supply not only extends the service area of the system but also mitigates the difficulty of finding a public bike or a parking slot.

## Supporting Information

S1 Fig Comparisons of the number of pickups and the number of returns at stations on weekdays and weekends. (1) The points represent each of bike stations, and blue and red symbols for weekdays and weekends respectively. (2)Y axis and X axis, represent the number of pickups and returns respectively. (3) Figs A represent the daily use, Figs B represent the hourly use during morning peak hours, and Figs C represent the hourly use during evening peak hours.
(TIF)

S1 File This file (Zip format) contains the data underlying the findings. (ZIP)

## References

[1] Krizek KJ, Barnes G, Thompson K. Analyzing the Effect of Bicycle Facilities on Commute Mode Share over Time. J Urban Plan Dev-ASCE. 2009;135(2):66–73.

[2] Pucher J, Dill J, Handy S. Infrastructure, programs, and policies to increase bicycling: An international review. Prev Med. 2010;50:S106–S25. 10.1016/j.ypmed.2009.07.028 19765610

[3] Keijer MJN, Rietveld P. How do people get to the railway station? The Dutch experience. Transportation Planning and Technology. 2000;23(3):215–35.

[4] Martens K. Promoting bike-and-ride: The Dutch experience. Transp Res Pt A-Policy Pract. 2007;41(4):326–38.

[5] DeMaio P. Bike-sharing:History, Impacts, Models of Provision, and Future. Public Transportation. 2009;12:16.

[6] Shaheen S, Guzman S, Zhang H. Bikesharing in Europe, the Americas, and Asia Past, Present, and Future. Transp Res Record. 2010;159–67.

[7] Fishman E, Washington S, Haworth N. Bike Share: A Synthesis of the Literature. Transp Rev. 2013;33(2):148–65.

[8] O’Brien O, Cheshire J, Batty M. Mining bicycle sharing data for generating insights into sustainable transport systems. Journal of Transport Geography. 2014;34:262–73.

[9] ITDP-China. China Bikesharing. Available from: http://www.publicbike.net/defaulten.aspx.

[10] Vogel M, Hamon R, Lozenguez G, Merchez L, Abry P, Barnier J, et al From bicycle sharing system movements to users: a typology of Vélo’v cyclists in Lyon based on large-scale behavioural dataset. Journal of Transport Geography. 2014;41:280–91.

[11] Borgnat P, Abry P, Flandrin P, Robardet C, Rouquier JB, Fleury E. SHARED BICYCLES IN A CITY: A SIGNAL PROCESSING AND DATA ANALYSIS PERSPECTIVE. Adv Complex Syst. 2011;14(3):415–38.

[12] Froehlich J, Neumann J, Oliver N, editors. Sensing and Predicting the Pulse of the City through Shared Bicycling. 21st International Joint Conference on Artificial intelligence; 2009; Pasadena, California, USA.

[13] Kaltenbrunner A, Meza R, Grivolla J, Codina J, Banchs R. Urban cycles and mobility patterns: Exploring and predicting trends in a bicycle-based public transport system. Pervasive and Mobile Computing. 2010;6(4):455–66.

[14] Vogel P, Greiser T, Mattfeld DC. Understanding Bike-Sharing Systems using Data Mining: Exploring Activity Patterns. Procedia—Social and Behavioral Sciences. 2011;20:514–23.

[15] Beecham R, Wood J. Exploring gendered cycling behaviours within a large-scale behavioural data-set. Transportation Planning and Technology. 2014;37(1):83–97.

[16] Zhao J, Wang J, Deng W. Exploring bikesharing travel time and trip chain by gender and day of the week. Transportation Research Part C: Emerging Technologies. 2015;58, Part B:251–64.

[17] Zhou X. Understanding Spatiotemporal Patterns of Biking Behavior by Analyzing Massive Bike Sharing Data in Chicago. PLoS ONE. 2015;10(10):e0137922 10.1371/journal.pone.0137922 26445357 PMC4596835

[18] Fuller D, Sahlqvist S, Cummins S, Ogilvie D. The impact of public transportation strikes on use of a bicycle share program in London: Interrupted time series design. Prev Med. 2012;54(1):74–6. 10.1016/j.ypmed.2011.09.021 22024219 PMC3821000

[19] Jäppinen S, Toivonen T, Salonen M. Modelling the potential effect of shared bicycles on public transport travel times in Greater Helsinki: An open data approach. Applied Geography. 2013;43:13–24.

[20] Jensen P, Rouquier J-B, Ovtracht N, Robardet C. Characterizing the speed and paths of shared bicycle use in Lyon. Transportation Research Part D: Transport and Environment. 2010;15(8):522–4.

[21] Goodman A, Cheshire J. Inequalities in the London bicycle sharing system revisited: impacts of extending the scheme to poorer areas but then doubling prices. Journal of Transport Geography. 2014;41.

[22] Lathia N, Ahmed S, Capra L. Measuring the impact of opening the London shared bicycle scheme to casual users. Transportation Research Part C: Emerging Technologies. 2012;22:88–102.

[23] Buck D, Buehler R. Bike Lanes and Other Determinants of Capital Bikeshare Trips. Transportation Research Board 91st Annual Meeting; Washington DC: Transportation Research Board; 2012.

[24] Daddio DW. Maximizing bicycle sharing: an empirical analysis of capital bikeshare usage. Master Thesis: University of North Carolina at Chapel Hill; 2012. Available from: http://rethinkcollegepark.net/blog/wp-content/uploads/2006/07/DaddioMP_Final-Draft.pdf

[25] El-Assi W, Salah Mahmoud M, Nurul Habib K. Effects of built environment and weather on bike sharing demand: a station level analysis of commercial bike sharing in Toronto. Transportation. 2015;10.1007/s11116-015-9669-z:1–25.

[26] Faghih-Imani A, Eluru N. Analysing bicycle-sharing system user destination choice preferences: Chicago’s Divvy system. Journal of Transport Geography. 2015;44:53–64.

[27] Faghih-Imani A, Eluru N. Incorporating the impact of spatio-temporal interactions on bicycle sharing system demand: A case study of New York CitiBike system. Journal of Transport Geography. 2016;54:218–27.

[28] Faghih-Imani A, Eluru N, El-Geneidy AM, Rabbat M, Haq U. How land-use and urban form impact bicycle flows: evidence from the bicycle-sharing system (BIXI) in Montreal. Journal of Transport Geography. 2014;41:306–14.

[29] Gonzalez F, Melo-Riquelme C, de Grange L. A combined destination and route choice model for a bicycle sharing system. Transportation. 2016;43(3):407–23.

[30] Nair R, Miller-Hooks E, Hampshire RC, Bušić A. Large-Scale Vehicle Sharing Systems: Analysis of Vélib'. International Journal of Sustainable Transportation. 2013;7(1):85–106.

[31] Rixey R. Station-level forecasting of bikesharing ridership: Station Network Effects in Three U.S. Systems. Transp Res Record. 2013;46–55.

[32] Wang X, Lindsey G, Schoner JE, Harrison A. Modeling Bike Share Station Activity: Effects of Nearby Businesses and Jobs on Trips to and from Stations. J Urban Plan Dev. 2016;142(1):04015001.

[33] Corcoran J, Li T, Rohde D, Charles-Edwards E, Mateo-Babiano D. Spatio-temporal patterns of a Public Bicycle Sharing Program: the effect of weather and calendar events. Journal of Transport Geography. 2014;41:292–305.

[34] Gebhart K, Noland RB. The impact of weather conditions on bikeshare trips in Washington, DC. Transportation. 2014;41(6):1205–25.

[35] Zhao J, Deng W, Song Y. Ridership and effectiveness of bikesharing: The effects of urban features and system characteristics on daily use and turnover rate of public bikes in China. Transport Policy. 2014;35(0):253–64.

[36] Dell'Amico M, Iori M, Stefano N, Stützle T. A Destroy and Repair Algorithm for the Bike sharing Rebalancing Problem. Comput Oper Res. 2016;

[37] Kadri A, Kacem I, Labadi K. A branch-and-bound algorithm for solving the static rebalancing problem in bicycle-sharing systems. Computers & Industrial Engineering. 2016;

[38] Neumann-Saavedra BA, Vogel P, Mattfeld DC. Anticipatory Service Network Design of Bike Sharing Systems. Transportation Research Procedia. 2015;10:355–63.

[39] ZhongshanStatisticsBureau. Zhongshan Statistic Yearbook 2014. Available from: http://www.zsda.gov.cn/plus/php_dq_zhishu.php.

[40] Frade I, Ribeiro A. Bicycle Sharing Systems Demand. Procedia—Social and Behavioral Sciences. 2014;111:518–27.

[41] Corcoran J, Li T. Spatial analytical approaches in public bicycle sharing programs. Journal of Transport Geography. 2014;41:268–71.

