# Supplementary figures and images for: Expanding Bicycle-Sharing Systems: Lessons Learnt from an Analysis of Usage
Source: PLoS One. 2016 Dec 15;11(12):e0168604. doi: 10.1371/journal.pone.0168604 (PMC5158202; doi:10.1371/journal.pone.0168604)

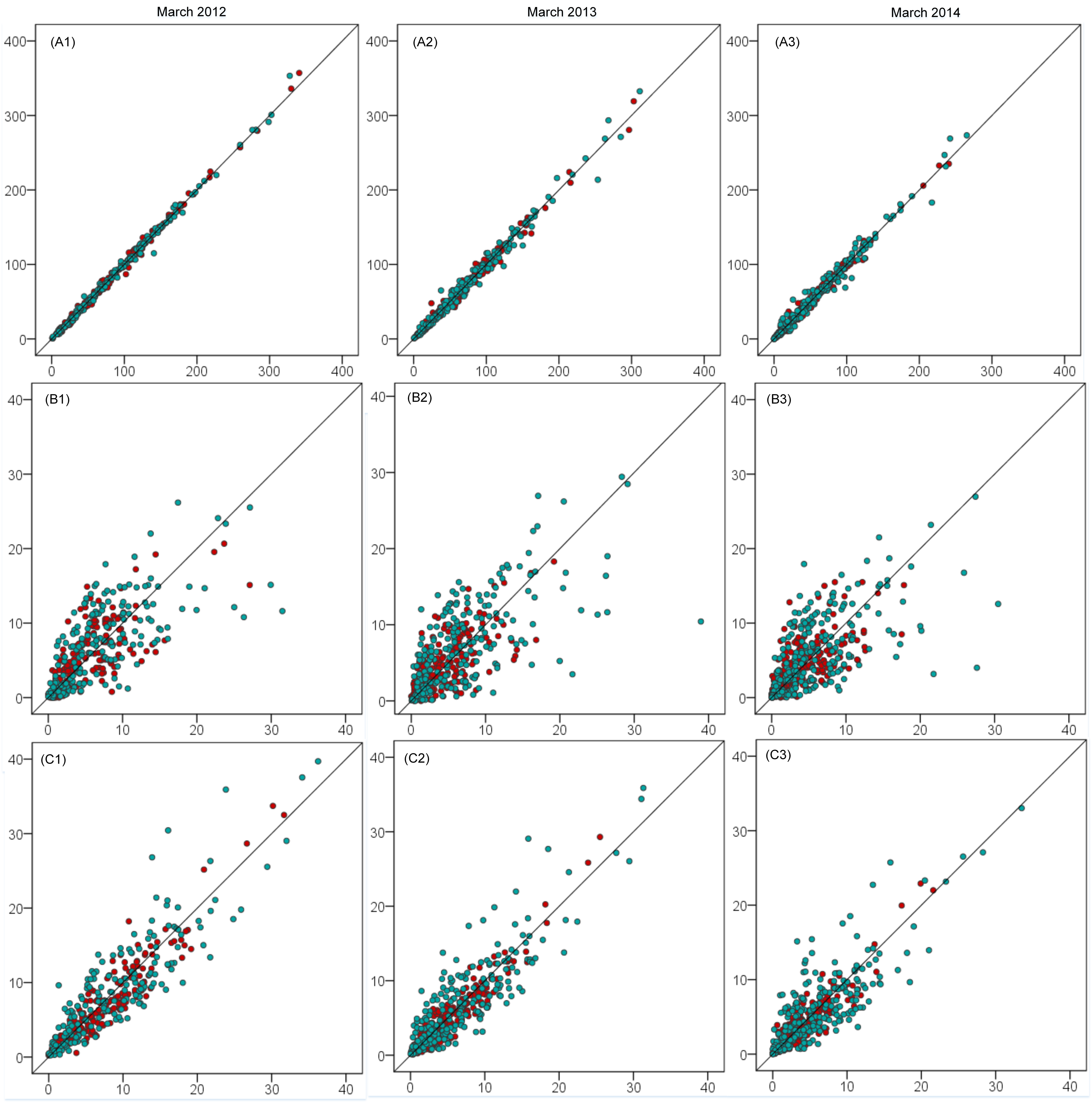

Supplement: S1 Fig — (1) The points represent each of bike stations, and blue and red symbols for weekdays and weekends respectively. (2)Y axis and X axis, represent the number of pickups and returns respectively. (3) Figs A represent the daily use, Figs B represent the hourly use during morning peak hours, and Figs C represent the hourly use during evening peak hours. (TIF) [file pone.0168604.s001.tif]
